# Efficacy of a “provisional incision” for longitudinal flap division after free-flap surgery^✰^

**DOI:** 10.1016/j.jpra.2022.07.003

**Published:** 2022-07-30

**Authors:** Y. Izawa, H. Murakami, T. Shirakawa, M. Nishida, K. Futamura, Y. Kobayashi, Y. Tsuchida

**Affiliations:** a Department of Orthopedic Trauma Center, Sapporo Higashi Tokushukai Hospital, Kita 33 jou Higashi 14 chome 3-1, Higashiku, Sapporo, Hokkaido, Japan; b Department of Trauma Center, Shonan Kamakura General Hospital, 1370-1, Okamoto, Kamakura-shi, Kanagawa, Japan; c Department of Orthopaedic Surgery, Surgical Science, Tokai University School of Medicine, 143 Shimokasuya, Isehara, Kanagawa, 259-1193, Japan

**Keywords:** Open fracture, Free-flap surgery, Flap necrosis, Provisional incision

## Abstract

Fix and flap surgery for severe open limb fractures is already a standard treatment. In cases where the fracture is complicated or accompanied by bone defects, secondary surgery is required for fracture sites covered with a myocutaneous flap after the soft tissue condition has stabilized. We applied the delayed procedure concept used for distant flaps and attempted to prevent postoperative myocutaneous flap necrosis by performing a provisional incision prior to the longitudinal incision of the flap. We report the course of five cases of the longitudinal division of the myocutaneous flap using “provisional incision” after free-flap surgery for severe open fracture and verify its usefulness. In this case series, five patients with severe open limb fractures treated from 2020 to 2021 who underwent longitudinal incision of the myocutaneous flap using provisional incision after free-flap surgery were included. The types of flaps used for soft tissue reconstruction in the acute phase, the reasons for the need for secondary surgery, the period from soft tissue reconstruction to additional surgery, and the healing status of soft tissue after secondary surgery were all investigated retrospectively. The types of flaps used for soft tissue reconstruction were latissimus dorsi myocutaneous flap in four cases and anterolateral thigh flap in one case. The breakdown of secondary surgery was osteosynthesis in one case, plate removal in one case, and bone cement removal and autologous bone grafting in three cases. The period from soft tissue reconstruction to secondary surgery ranged from 6 weeks to 4 months. In all cases, the wound healed without necrosis of the myocutaneous flap. For the treatment of severe open limb fractures, longitudinal division of the myocutaneous flap using “provisional incision” is a safer approach to the necessary secondary surgery and reduces the possibility of necrosis of the flap.

## Introduction

**Fig. 1:**
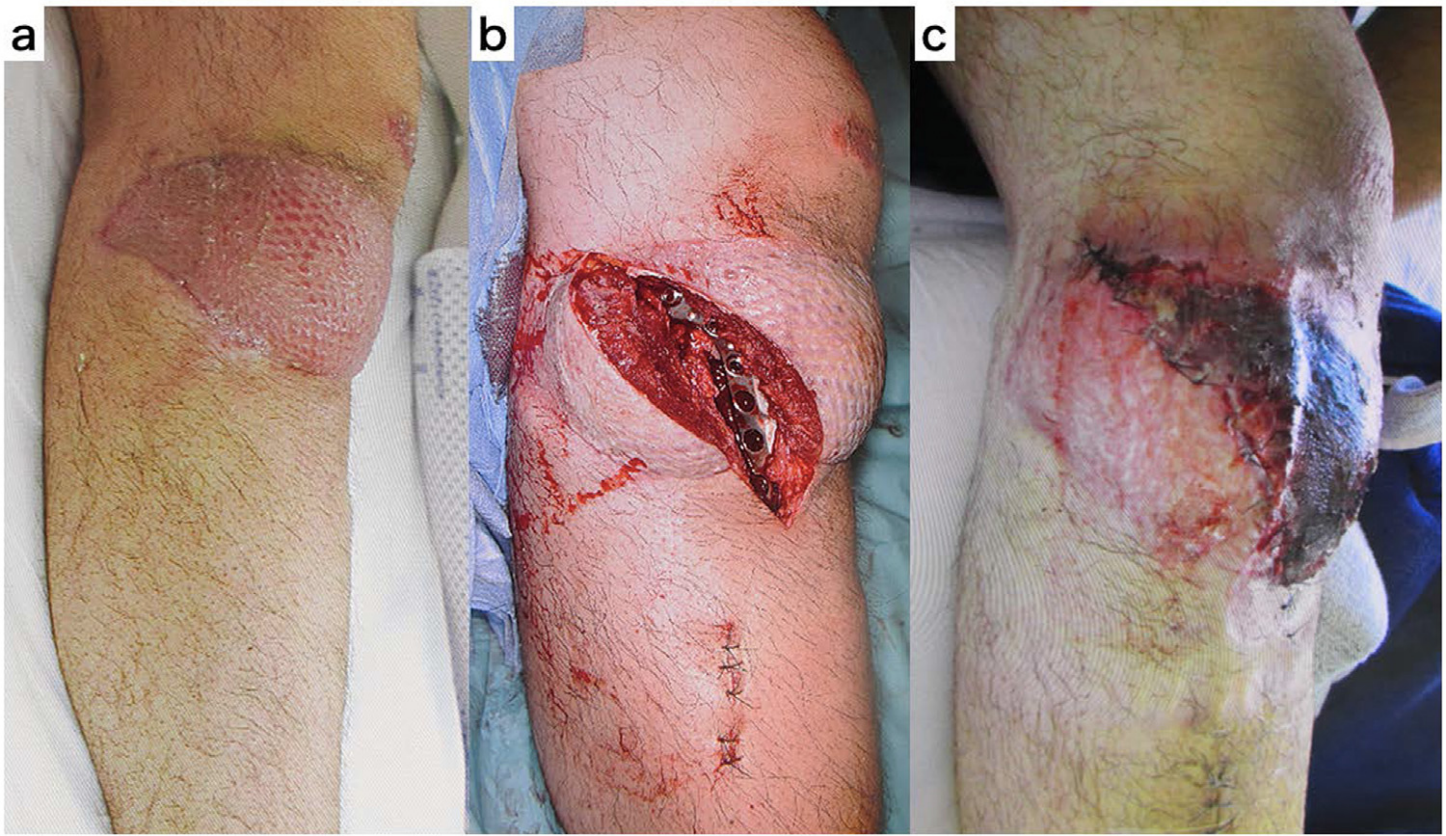
The case presentation of flap necrosis of the distal half after longitudinal division of the muscle flap. a. Seven months after surgery of lateral gastrocnemius muscle flap. b. The muscle flap was longitudinally divided, and additional osteosynthesis was performed on the lateral side of the proximal tibia. c. The distal half of the muscle flap was necrotic after surgery.

Fix and flap surgery for severe open limb fractures is already a standard treatment.^1^^,^^2^ In cases where the fracture is complicated or accompanied by bone defects, secondary surgery, such as additional osteosynthesis, implant removal, and bone grafting, is required for fracture sites covered with a myocutaneous flap after the soft tissue condition has stabilized.^3^^,^^4^ As an approach for performing secondary surgery, the fracture site is usually entered from the flap margin; however, it is often more effective to make a longitudinal incision in the flap just above the fracture. However, even in cases where the skin flaps and muscle flaps have survived without problems, we often encounter cases in which necrosis occurs at the wound edge opposite to the vascular pedicle after performing a longitudinal incision in the skin flaps and muscle flaps (Fig. 1). We applied the delayed procedure concept used for distant flaps and attempted to prevent postoperative myocutaneous flap necrosis by performing a provisional incision prior to the longitudinal incision of the flap. We report the course of five cases of the longitudinal division of the myocutaneous flap using “provisional incision” after free-flap surgery for severe open fracture and verify its usefulness.

## Patients and methods

In this case series, five patients with severe open limb fractures treated at our hospital from 2020 to 2021 who underwent longitudinal incision of the myocutaneous flap using provisional incision after free-flap surgery were included. The patients were three men and two women whose ages ranged from 19 to 86 years, with an average age of 46.2 years. The area of injury were the left hand in one case, left lower extremity in one case, and right lower extremity in three cases. Two patients were injured by being caught in a machine, and three patients were injured in a traffic accident. The types of flaps used for soft tissue reconstruction in the acute phase, the reasons for the need for secondary surgery, the period from soft tissue reconstruction to additional surgery, and the healing status of soft tissue after secondary surgery were all investigated retrospectively.

### Operative technique

**Fig. 2:**
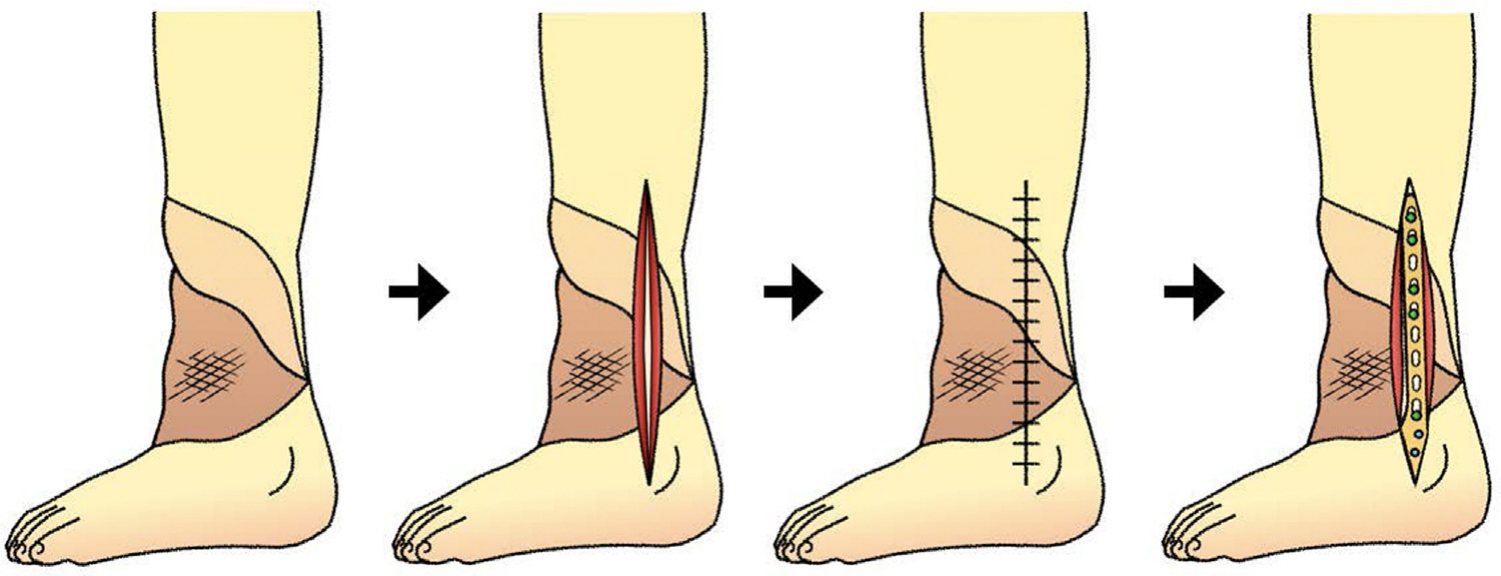
Schematic representation of the provisional incision after surgery of the free latissimus dorsi myocutaneous flap. As provisional incision, myocutaneous flap is devided longitudinally to the bone at once without deep peeling, and the wound is sutured roughly. About one week later, deep peeling and the necessary operation are performed from the same incision.

The procedure for surgery using a “provisional incision” is as follows. First, the most effective incision line for the secondary surgery shoud be designed. The incision line should be in the long axis direction of the limbs, regardless of the arrangement of the vascular pedicle and the fiber direction of the myocutaneous flap so that the myocutaneous flap is completely divided. Using a scalpel, make an incision at the bone layer, implant, and bone cement all at once, confirming that there is bleeding from both sides of the longitudinally split flap. At this point, only the incision is made, and no deep peeling operation is performed. The surface layer of the incision is gently sutured with a nylon thread to close the wound. After waiting for 1 week, deep peeling from the same incision line and any necessary operations shoud be performed (Fig. 2).

## Results

The types of flaps used for soft tissue reconstruction were the latissimus dorsi myocutaneous flap in four cases and the anterolateral thigh flap in one case. The breakdown of secondary surgery was osteosynthesis in one case, plate removal in one case, and bone cement removal and autologous bone grafting in three cases. The period from soft tissue reconstruction to secondary surgery ranged from 6 weeks to 4 months. In all cases, the wound healed without necrosis of the myocutaneous flap (Table 1).

**Table 1:** Demographic data, type of the surgery at acute phase, timing, and breakdown of the secondary surgery.

Case	Age	Sex	Injury	Surgery at acute phase		Period from flap surgery to secondary surgery	Secondary surgery	Wound healing after second surgery
				osteosynthesis	Flap			
1	33	Male	Open wrist fracture G-A Type 3C	Wrist: plating	LD	6 weeks	Remove plate	No necrosis
2	19	Female	Open lower leg fracture G-A Type 3B	Tibia: plating fibula: neglect	LD	3 months	Plating for fibula	No necrosis
3	44	Male	Open lower leg fracture G-A Type 3B	Tibia: plating filling bone cement	LD	3 months	Remove cement exchange to nail	No necrosis
4	86	Female	Pilon fracture infection after surgery	Tibia: plating filling bone cement	LD	3 months	Remove cement bone grafting	No necrosis
5	49	Male	Open foot fracture G-A Type 3B	1st metatarsal: plating filling bone cement	ALT	4 months	Remove cement bone grafting	No necrosis

G-A: Gustilo-Anderson classification.

LD: Free latissimus dorsi myocutaneous flap.

ALT: Free anterolateral thigh flap.

### Case reports

#### Case 1

**Fig. 3:**
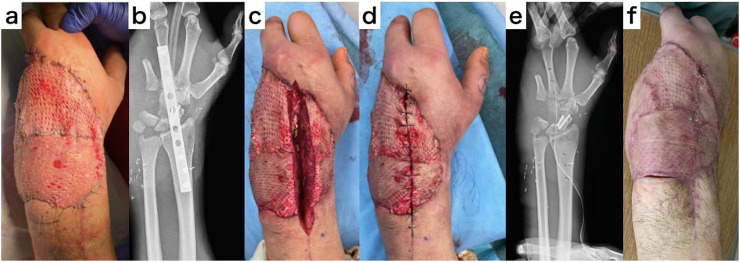
Treatment course of case 1. a. Appearance before secondary surgery. b. X-ray before secondary surgery. c. Appearance after provisional incision. d. Appearance after rough suture. e. X-ray after secondary surgery. f. Appearance after wound healing.

A 33-year-old man presented with an open wrist fracture Gustilo-Anderson classification Type 3C on his left hand, which was caught in a conveyor belt when he was working. Revascularization was performed on the first day, and fix and flap surgery was performed on the seventh day after injury. A 1/3 tubular plate was used to fix the joint from the metaphysis of the radius to the metacarpal bone of the middle finger, and the ruptured ulnar artery stump was used as a recipient to reconstruct the soft tissue with a free latissimus dorsi myocutaneous flap. After the soft tissue condition stabilized, we planned the removal of the plate for wrist movement. Five weeks after the flap surgery, a longitudinal incision was made in the myocutaneous flap just above the plate. One week later, the plate was removed, and the wound was closed. The wound healed without any trouble in the longitudinally divided muscle flap (Fig. 3).

#### Case 2

**Fig. 4:**
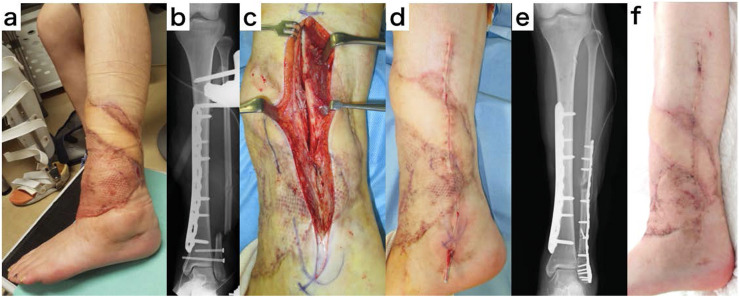
Treatment course of case 2. a. Appearance before secondary surgery. b. X-ray before secondary surgery. c. Appearance after provisional incision. d. Appearance after rough suture. e. X-ray after secondary surgery. f. Appearance after wound healing.

A 19-year-old woman was injured in a car accident and brought to a different hospital. She was presented with an open lower leg fracture Gustilo-Anderson classification Type 3B, with a 20 × 25-cm soft tissue defect at the time of transfer to our hospital. Fix and flap surgery was performed on the tenth day after injury. The tibial diaphyseal fracture was fixed with a medial locking plate and then covered with a free latissimus dorsi myocutaneous flap with the posterior tibial artery as the recipient. Since the required coverage area was expected to be large, we planned to perform osteosynthesis after the soft tissue condition was stabilized without osteosynthesis of the fibula fracture. Three months after the flap surgery, a longitudinal incision was made in the myocutaneous flap just above the fracture of the fibula. Ten days later, osteosynthesis with a lateral locking plate was performed, and the wound was closed. The wound healed without any trouble in the longitudinally divided muscle flap (Fig. 4).

#### Case 3

A 44-year-old man was injured in a motorcycle accident and brought to a different hospital. He was presented with an open lower leg fracture Gustilo-Anderson classification Type 3B, with a tibial bone defect of approximately 75 mm at the time of transfer to our hospital. We planned to perform the fix followed by flap and bone length-preserving treatment. On the fifth day after the injury, osteosynthesis was performed with a plate while maintaining the bone length of the tibia, and the defect was filled with bone cement containing antibiotics. Soft tissue reconstruction was performed on the tenth day after injury using a free latissimus dorsi myocutaneous flap and serratus anterior muscle flap with the posterior tibial artery as the recipient. After the soft tissue condition had stabilized, we planned to insert an intramedullary nail and replace the bone cement. Ten weeks after the flap surgery, a longitudinal incision was made in the myocutaneous flap just above the bone cement in front of the lower leg. One week later, the plate was removed, an intramedullary nail was inserted, and the bone cement was replaced. The wound healed without any trouble in the longitudinally divided muscle flap.

#### Case 4

An 86-year-old woman presented with a pilon fracture when she was hit by a car while walking and was brought to a different hospital. Plate fixation was performed, but skin necrosis was observed on the medial and lateral sides of the lower leg after surgery, and soft tissue reconstruction was necessary. Thus, the patient was transferred to our hospital. Soft tissue defects measuring 8 × 6 and 12 × 7 cm were found on the medial and lateral sided of the lower leg. On the 18th day of injury, both plates were removed, and bone cement containing antibiotics was placed for bone defects in the tibia. Fix and flap surgery was performed on the 25th day after injury. The tibia was fixed with a medial locking plate, and a free latissimus dorsi myocutaneous flap surgery was performed with the posterior tibial artery as the recipient. Two months after the flap surgery, bone cement removal and autologous bone grafting were planned as the 2nd stage treatment using the Masquelet method. A prior longitudinal incision was made at the boundary between the skin flap and muscle flap just above the bone cement. Twelve days later, the cement was removed, and bone grafting was performed. The wound healed without any complications in the longitudinally divided flap.

#### Case 5

A 49-year-old man presented with an open-foot fracture Gustilo-Anderson classification Type 3B on his right leg due to a machine-related accident. Fix and flap surgery was performed on the seventh day after injury. For the first metatarsal bone, the defect was reconstructed with bone cement and fixed with a plate, and soft tissue was reconstructed with a free anterolateral thigh flap with the dorsalis pedis artery as the recipient. Four months after the flap surgery, when the soft tissue had stabilized, a prior longitudinal incision of approximately 10 cm was made just above the bone cement in the first metatarsal bone. One week later, the bone cement was removed, replaced with autologous bone, and fixed again with a plate. The wound healed without any complications in the longitudinally divided flap.

## Discussion

To our knowledge, there is currently no literature demonstrating the intentional use of longitudinally divided myocutaneous flaps for secondary surgery. In the case of arterial flaps and muscle flaps, necrosis may occur by cutting off its vascular pedicle.^5^^,^^6^ However, distant flaps can be dissected from the donor site at approximately 3 weeks after soft tissue transplantation and will survive with blood flow from the recipient bed and surrounding tissue. Considering that the cross leg free latissimus dorsi muscle flap can dissect the vascular pedicle about 3–4 weeks after transplantation, the latissimus dorsi muscle flap can also survive by blood flow from the recipient bed and surrounding tissue. Therefore, necrosis can be avoided if the blood flow from the recipient bed and the surrounding tissue is sufficient, despite blocking the blood flow from the vascular pedicle to the distal side by longitudinal division of the myocutaneous flap.^7^, ^8^, ^9^

However, in one of our cases, the myocutaneous flap did not survive solely on blood flow from the recipient bed and surrounding tissue as necrosis was observed on the distal side of the longitudinal division and osteosynthesis was performed. Therefore, it is necessary to devise a method to prevent flap necrosis when performing longitudinal division of the myocutaneous flap; herein, the use of a provisional incision was considered. In this method, only the incision of the flap was performed; the deep layer was not peeled off, and angiogenesis was expected from the surroundings by delay phenomenon.^10^^,^^11^ After 1 week, the inflow of blood vessels from the surroundings, the expansion of blood vessel diameter, and the increase in blood flow velocity were observed. This operation is a safer approach to the necessary secondary surgery as it reduces the possibility of necrosis of the flap on the distal side, where blood flow from the pedicle side is cut off.

## Conclusion

In all five cases of severe open fractures with longitudinal division of the myocutaneous flap using “provisional incision” after free-flap surgery, necrosis of the flap did not occur. Longitudinal division of the myocutaneous flap using “provisional incision” is a safer and effective approach to the necessary secondary surgery, and it reduces the possibility of necrosis of the flap.

## Ethical approval

This study was approved by the institutional review board (SKEC-21–26).

## Informed consent

Informed consent was obtained from all individuals participants included in the study.

## Declaration of Competing Interest

None.

## Funding

This research did not receive any specific grant from funding agencies in the public, commercial, or not-for-profit sectors.
